# Universal protective and risk factors of mental health during the COVID-19 pandemic: The role of compassion and fears of compassion

**DOI:** 10.1192/j.eurpsy.2023.885

**Published:** 2023-07-19

**Authors:** M. Matos, C. A. C.-1. Consortium

**Affiliations:** 1 Center for Research in Neuropsychology and Cognitive and Behavioral Intervention (CINEICC), University of Coimbra, Coimbra; 2 Multinational, Multinational, Multinational, Portugal

## Abstract

**Introduction:**

The COVID-19 pandemic has had an unprecedented detrimental impact on mental health in people around the world. It is therefore important to examine factors that may buffer or heighten the risk of mental health problems in this context.

**Objectives:**

The current study explores the buffering effects of different flows of compassion (for self, for others, from others) and the magnifying effects of fears of compassion on the impact of perceived threat of COVID-19 on indicators of mental health and psychosocial wellbeing.

**Methods:**

4057 adult participants collected from the general community population across 21 countries from Europe, Middle East, North America, South America, Asia and Oceania, completed self-report measures of perceived threat of COVID-19, compassion, fears of compassion, social safeness, loneliness, depression, anxiety, stress, posttraumatic stress and growth.

**Results:**

Self-compassion moderated the impact of perceived threat of COVID-19 on depression, anxiety and stress, whereas compassion from others moderated the effects of fears of COVID-19 on social safeness. Fears of compassion moderated the impact of perceived threat of COVID-19 on psychological distress. Only fears of compassion from others moderated the effects of fears of COVID-19 on social safeness. Furthermore, social connection (compassion and social safeness) predicted higher post-traumatic growth and traumatic stress, whereas social disconnection (fears of compassion and loneliness) predicted increased traumatic symptoms only. Social connection heightened the impact of perceived threat of COVID-19 on post-traumatic growth, while social disconnection weakened this impact. Social disconnection magnified the impact of the perceived threat of COVID-19 on traumatic stress. The effects were consistent across countries.

**Conclusions:**

Our findings highlight the universal protective role of compassion and social connection in promoting resilience and buffering against the harmful effects of the COVID-19 pandemic on mental health and psychosocial wellbeing. Furthermore, our results reveal that fears of compassion have a magnifying effect on the damaging impact of the COVID-19 pandemic on mental health.

**Disclosure of Interest:**

None Declared

